# Detection of novel loci involved in non-seed-shattering behaviour of an *indica* rice cultivar, *Oryza sativa* IR36

**DOI:** 10.1007/s00438-023-02027-z

**Published:** 2023-05-17

**Authors:** Shohei Sugiyama, Motoki Sakuta, Yuki Tsujimura, Yudai Yamaguchi, Than Myint Htun, Chizuru Inoue, Koji Numaguchi, Takashige Ishii, Ryo Ishikawa

**Affiliations:** 1 Laboratory of Plant Breeding, Graduate School of Agricultural Science, Kobe University, 1-1 Rokkodai-cho, Nada-ku, Kobe, 657-8501 Japan; 2 Present Address: Department of Plant Breeding, Physiology and Ecology, Yezin Agricultural University, Yezin, Nay Pyi Taw, 15013 Myanmar

**Keywords:** Rice (*Oryza sativa*), *Oryza rufipogon*, Seed shattering, *sh4*, *qSH3*, Domestication

## Abstract

Asian rice (*Oryza sativa*) was domesticated from *O. rufipogon*, and reduced seed-shattering behaviour was selected to increase yields. Two seed-shattering loci, *qSH3* and *sh4*, are involved in reducing seed shattering in both *japonica* and *indica* rice cultivars, while *qSH1* and *qCSS3* are likely specific to *japonica* cultivars. In *indica* cultivars, *qSH3* and *sh4* fail to explain the degree of seed shattering, as an introgression line (IL) of *O. rufipogon* W630 carrying domesticated alleles at *qSH3* and *sh4* still showed seed shattering. Here we analysed differences in seed-shattering degree between the IL and the *indica* cultivar IR36. The values for grain detachment in the segregating population between the IL and IR36 were continuous. Based on QTL-seq analysis using the BC_1_F_2_ population between the IL and IR36, we detected two novel loci, *qCSS2* and *qCSS7* (*QTLs for the Control of Seed Shattering in rice on chromosomes 2 and 7*), which contributed to the reduced seed shattering in IR36. We further investigated the genetic interaction of *qCSS2* and *qCSS7* under the presence of *qSH3* and *sh4* mutations in *O. rufipogon* W630 and found that IL carrying IR36 chromosomal segments covering all four loci are required to explain seed-shattering degree in IR36. Since *qCSS2* and *qCSS7* were not detected in previous studies on seed shattering in *japonica*, their control may be specific to *indica* cultivars. Therefore, they are important to understanding the history of rice domestication as well as to adjusting the seed-shattering degree of *indica* cultivars to maximise their yield.

**Supplementary Information:**

The online version contains supplementary material available at 10.1007/s00438-023-02027-z.

## Introduction

A reduction in seed shattering is a well-known phenotypic change observed in many crops (Doebley et al. 2006). Wild plants shatter their seeds soon after maturation for more efficient propagation, but early farmers selected plants with reduced seed shattering to improve harvest efficiency. The more stable yield provided by the loss of seed shattering allowed for more efficiently organised agricultural systems, an important factor leading to the development of human civilisation. Rice (*Oryza sativa* L.) is a vital staple crop worldwide, together with wheat and maize, and is domesticated from the wild rice *O. rufipogon*, which is widely distributed in tropical Asian countries (Khush 1997). During its domestication, several important characteristics were altered to make it more convenient for human use (Ishikawa et al. 2020).

Notably, inhibition of abscission layer formation is associated with reduced seed-shattering behaviour, and genetic analyses have revealed several loci involved in a loss of seed shattering based on natural variations in the degree of seed shattering in wild and cultivated rice species. A major locus of *sh4* was identified by a quantitative trait locus (QTL) analysis of the degree of seed shattering of the F_2_ segregating population between the wild rice *O. nivara* (an annual form of *O. rufipogon*) and *O. sativa indica* rice (Li et al. 2006). The mutation at *sh4* was caused by a single-nucleotide polymorphism (SNP) in the gene encoding the MYB transcription factor. All the cultivated rice investigated for their genotype at *sh4* carried the mutation, indicating it played an important role in reducing seed shattering during rice domestication. Another locus of *qSH1* was identified by QTL analysis using the F_2_ segregating population between two cultivated rice varieties, *O. sativa* Nipponbare and Kasalath (Konishi et al. 2006). The mutation was found to be an SNP in the regulatory region of the gene encoding a transcription factor, similar to *Arabidopsis REPLUMLESS*, and the mutation was found among *japonica* rice cultivars. The seed-shattering locus *qSH3* was originally detected by QTL analysis (Onishi et al. 2007) and was further confirmed by the QTL analysis of the degree of seed shattering using the segregating population between *O. sativa* Nipponbare and *O. rufipogon* (Htun et al. 2014). The causal mutation at *qSH3* was revealed to be an SNP in the previously identified gene of *OsSh1*, a homologue of the sorghum seed-shattering gene (Lin et al. 2012; Ishikawa et al. 2022). Interestingly, introgression lines (IL) carrying the domesticated allele at *qSH3* in the wild genetic background showed complete seed-shattering behaviour as the wild rice *O. rufipogon*, suggesting that the single mutation at *qSH3* in wild rice was insufficient to reduce seed shattering, and which is also observed for *sh4* (Ishikawa et al. 2022). However, the ILs having mutations at both *qSH3* and *sh4* slightly inhibited the abscission layer formation, resulting in reduced seed-shattering behaviour (Inoue et al. 2015; Ishikawa et al. 2022). In our previous study, we reported *qCSS3*, a novel locus involved in reducing seed shattering in the *japonica* cultivar Nipponbare (Tsujimura et al. 2019). Studies of these loci suggested that a loss of seed shattering in cultivated rice can be established by the combination of variations at several loci with specific and common mutations among cultivars. Furthermore, we previously investigated the non-seed-shattering behaviour of the *indica* cultivar *O. sativa* IR36, one of the leading varieties grown in tropical Asia with semi-dwarf morphology and high resistance to major insects and disease (Spielmeyer et al. 2002; Ballini et al. 2007), and showed that *qSH3* and *sh4* were responsible for the reduced shattering phenotype (Ishikawa et al. 2017).

In this study, we aimed to understand the genetic loci involved in the reduced seed-shattering behaviour of IR36. We performed a QTL analysis for the degree of seed shattering on the segregating population between IR36 and wild rice IL carrying domesticated alleles at *qSH3* and *sh4*. The loci identified in this study were further evaluated to confirm their roles in regulating rice seed shattering.

## Material and methods

### Plant materials

An *indica* rice cultivar *O. sativa* cv. IR36, a *japonica* rice cultivar *O. sativa* cv. Nipponbare, and the wild rice accession of *O. rufipogon* acc. W630 originated from Myanmar were used in this study. By backcrossing with the W630, an IL carrying the Nipponbare chromosomal segments covering the *qSH3* and *sh4* loci in the genetic background of wild rice was produced and named as IL(*qSH3*-Npb, *sh4*-Npb). The IL(*qSH3*-Npb, *sh4*-Npb) was crossed with IR36, and the resulting F_1_ plant was further backcrossed with IR36. Among 116 BC_1_F_1_ plants grown in 2016, No. 112 with higher seed-shattering behaviour was selected (Supplementary Table 2), and their self-pollinated BC_1_F_2_ seeds were obtained. A total of 166 BC_1_F_2_ individuals were grown in pots at Kobe University, Japan in 2017, with short-day treatments to induce similar flowering times, and their seed-shattering behaviour was evaluated. A progeny test was also conducted using two lines with heterozygous chromosomal constitution at the locus detected in this study.

### Evaluation of the degree of seed shattering

To evaluate the degree of seed shattering, we measured the breaking tensile strength (BTS, gf: gram-force), which is the amount of force required to detach a grain from the pedicel, using a digital force gauge (FGP 0.5, Nidec-Shimpo Co., Japan). Approximately one month after heading, the BTS values of 75 seeds (25 randomly selected seeds from each of the three panicles) were measured, and their average BTS values were calculated.

### DNA extraction, bulking, and library construction for next-generation sequencing analysis

Bulked DNA samples were prepared for QTL-seq analysis as described in previous studies (Abe et al. 2012; Takagi et al. 2013). DNA was extracted from 100 mg of fresh rice leaves using the DNeasy Plant Mini Kit (QIAGEN Sciences, Germany). Then their concentrations were quantified using the Qubit® 3.0 Fluorometer and Qubit^®^ dsDNA BR Assay Kit (Life Technologies, Japan). Ten plants with the lowest and the highest BTS values were selected and genomic DNA from each plant was extracted and bulked in equal amounts into low- or high-BTS samples (L-bulk and H-bulk, respectively). The bulked samples and the IL(*qSH3*-Npb, *sh4*-Npb) as controls were subjected to whole-genome resequencing analysis using Illumina Hi-seq 2500 platform (paired-end 100).

### Read processing

The Illumina short reads obtained from the bulked and control samples, IL(*qSH3*-Npb, *sh4*-Npb) and IR36, were processed as follows. Raw reads were first subjected to adapter and quality trimming using PEAT (Li et al. 2015) and Trimmomatic (Bolger et al. 2014), respectively. The parameters of Trimmomatic were: LEADING: 20, TRAILING: 20, SLIDINGWINDOW: 10: 20 and MINLEN: 20. Trimmed reads were aligned to the Nipponbare reference genome sequence obtained from the Rice Annotation Database Project (http://rapdb.dna.affrc.go.jp) using BWA-MEM (Li 2013) and transformed into BAM files using SAMtools (Danecek et al. 2021). Then, PCR duplicates were removed using Picard (http://broadinstitute.github.io/picard/). Resulting BAM files were used for the investigation of introgressed chromosomal segments and QTL-seq analysis.

### Estimation of the chromosomal segments of Nipponbare and IR36

BAM files (PCR duplicates were removed) were processed into gVCFs using HaplotypeCaller function of GATK4 (Van der Auwera and O’Connor 2020). Each gVCF was combined into a VCF by GenomicsDBImport and GenotypeGVCFs functions of GATK4. SNP loci were selected, and quality filtered by VariantFiltration of GATK4 according to the following thresholds, QD < 5.0, QUAL < 30, SOR > 3.0, FS > 60.0, MQ < 50.0, MQRankSum < − 12.5 and ReadPosRankSum < − 8.0. SNP loci with missing data were further filtered using VCFtools (Danecek et al. 2011). Using the resulting VCF, we first estimated the chromosomal segments of Nipponbare that were introgressed in wild rice, *O. rufipogon* W630 genome using the Integrative Genomics Viewer (Robinson et al. 2011). We also investigated the IR36-homozygously fixed chromosomal regions in the BC_1_F_2_ population associated with backcrossing.

### Detection of novel seed-shattering loci by QTL-seq analysis

The BAM files of the bulked samples and the IL(*qSH3*-Npb, *sh4*-Npb) were subjected to QTL-seq Pipeline (Takagi et al. 2013; Sugihara et al. 2022). The SNP-index was calculated for all the SNP positions. The Δ(SNP-index) was then calculated by subtracting the SNP-index values of the L-bulk from those of the H-bulk. A sliding window analysis was applied by averaging the Δ(SNP-index) values within a 500 kb window size and a 25 kb increment. Regions with averaged Δ(SNP-index) values exceeding the 95% confidence intervals were regarded as statistically significant loci under the null hypothesis of no QTL (*P* < 0.05). The 95% confidence intervals under the null hypothesis were preliminary defined for each read depth and are implemented on the QTL-seq pipeline (Takagi et al. 2013). Simple sequence repeat (SSR) or indel markers were used to survey the genotypes at the detected loci (Supplementary Table 1).

### Morphological and histological analysis of abscission layer formation

A Leica Biosystems S6D microscope (Germany) was used to examine the abscission layer (axial images of detached spikelets), and MC170HD and the Leica Application Suite were used for photography (Leica Biosystems, Germany). Histological analysis of abscission layer formation was carried out using the pedicel tissue of grains before heading, as previously reported (Htun et al. 2014; Inoue et al. 2015). FAA solution (formaldehyde: acetic acid: 70% ethanol = 1:1:18 [volume ratio]) was used to fix the samples, followed by vacuum infiltration and preservation at 4 °C. They were dehydrated in an ethanol series (70%, 80%, 90% and 95% ethanol) for 2 days at each stage and then embedded in Technovit 7100 resin (Heraeus Kulzer, Germany), according to the manufacturer’s instructions. The samples were cut into 3-μm sections using an RM1215RT rotary microtome (Leica Biosystems, Germany), and toluidine blue O solution was used for staining. These sections were observed under a microscope and photographed with a digital camera using the imaging software, ToupView imaging software (× 86) (AmScope.com, USA).

## Results

### Characterisation of the seed-shattering behaviour of the IL(*qSH3*-Npb, *sh4*-Npb)

**Fig. 1:**
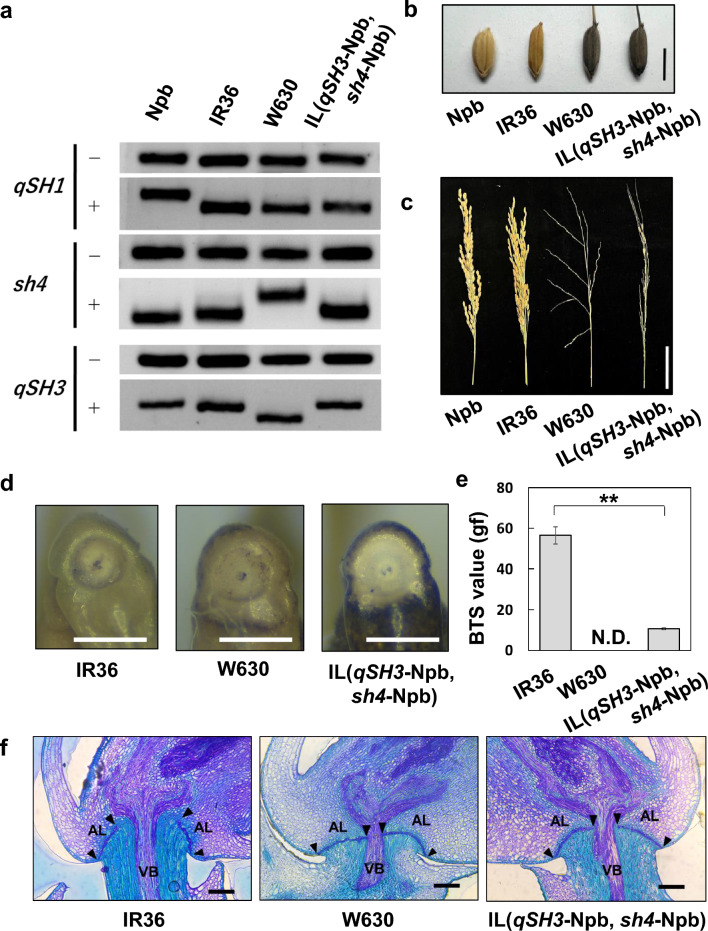
Characterisation of the introgression line (IL), IL(*qSH3*-Npb, *sh4*-Npb), carrying *O. sativa japonica* cv. Nipponbare (Npb) chromosomal segments covering *qSH3* and *sh4* loci in the genetic background of wild rice, *O. rufipogon* W630. **a** Genotyping at *qSH1*, *sh4*, and *qSH3* for *O. sativa japonica* cv. Nipponbare, *indica* cv. IR36, *O. rufipogon* acc. W630, and the IL(*qSH3*-Npb, *sh4*-Npb) based on the causal single-nucleotide polymorphisms (SNPs) detected by derived cleaved amplified polymorphic sequence (dCAPS) markers (Supplementary Table 1). − and + represent PCR fragments undigested and digested PCR fragments with enzymes, respectively. **b** Seeds of Nipponbare, IR36, W630, and IL(*qSH3*-Npb, *sh4*-Npb). Scale bar = 5 mm. **c** Panicle morphologies of Nipponbare, IR36, W630, and IL(*qSH3*-Npb, *sh4*-Npb) approximately one month after flowering. Scale bar = 5 cm. **d** A close view of the spikelet base in IR36, W630, and IL (from left to right). Scale bars = 1 mm. **e** The breaking tensile strength (BTS) values for IR36, W630, and IL(*qSH3*-Npb, *sh4*-Npb). Data are mean ± S.D. (n = 3). ** indicates *P* < 0.01 by an unpaired Student’s *t*-test. N.D., not determined owing to complete seed shattering. **f** Longitudinal sections of the spikelet base after seed detachment in IR36 (left), W630 (centre), and IL(*qSH3*-Npb, *sh4*-Npb) (right). *VB* vascular bundle. *AL* abscission layer. Black triangles indicate both edges of the abscission layer. Scale bars = 100 μm

As reported previously, IR36 carries non-functional alleles at the causal SNPs of both *qSH3* and *sh4*, and a functional allele at the causal SNP of *qSH1* (Ishikawa et al. 2017). We previously developed an IL between *O. sativa japonica* Nipponbare and *O. rufipogon* W630. The IL(*qSH3*-Npb, *sh4*-Npb) carries *O. sativa japonica* Nipponbare chromosomal segments covering both *qSH3* and *sh4* in the genetic background of wild rice, *O. rufipogon* W630 (Ishikawa et al. 2022). Here, we confirmed that the IL carries domesticated alleles at *qSH3* and *sh4*, with a functional allele at *qSH1* (Fig. 1a). The appearance of the seed and panicle of the IL was similar to that of wild rice, *O. rufipogon* W630 (Fig. 1b and c). First, we investigated the seed-shattering behaviour of the IL. Comparison of the morphology of the spikelet base for IR36, W630, and IL(*qSH3*-Npb, *sh4*-Npb) revealed similar detachment at the abscission zone (Fig. 1d), but the degree of seed shattering was different; IR36 showed a BTS of 56.5 ± 4.3 gf, while W630 completely shed seeds at maturation. Comparatively, IL(*qSH3*-Npb, *sh4*-Npb) had a detectable BTS value of 10.7 ± 0.5 gf, which is lower than that of IR36 (Fig. 1e). We also confirmed that the differences in the degree of seed shattering can be attributed to the degree to which abscission layer formation was inhibited (Fig. 1f). In agreement with the findings of our previous study (Ishikawa et al. 2022), an increase in the BTS value was proportionally associated with an increase in abscission layer inhibition. Based on the analysis of the seed-shattering behaviour of the IL(*qSH3*-Npb, *sh4*-Npb), novel seed-shattering loci may still contribute to reduced seed shattering in IR36.

### The degree of seed shattering in segregating populations obtained by crossing IR36 and IL(*qSH3*-Npb, *sh4*-Npb)

**Fig. 2:**
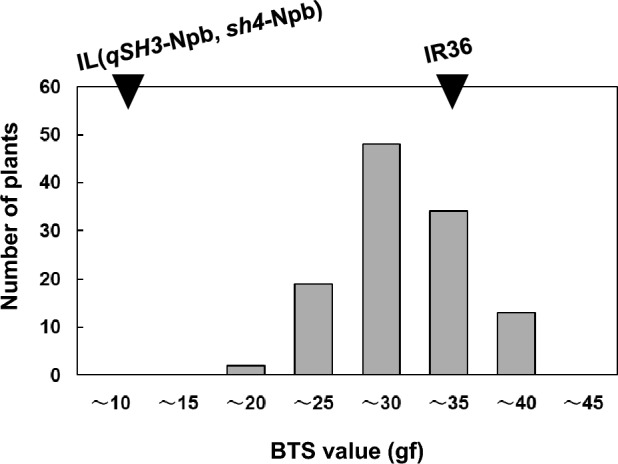
Frequency distribution of breaking tensile strength (BTS) values for 116 BC_1_F_1_ individuals between *Oryza* *sativa* IR36 and IL(*qSH3*-Npb, *sh4*-Npb). The average BTS values for the parent lines are indicated by black triangles. IR36: 34.3 ± 3.3 gf (n = 5) and the IL(*qSH3*-Npb, *sh4*-Npb), 11.6 ± 0.8 (n = 5) (mean ± SD). The BTS values for each BC_1_F_1_ individual are shown in Supplementary Table 2

**Fig. 3:**
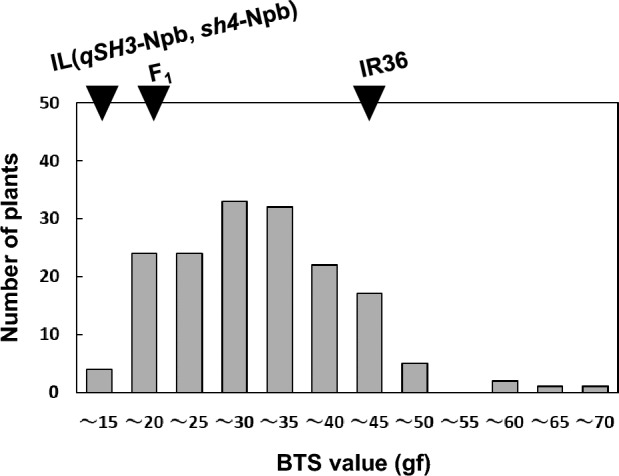
Frequency distribution of breaking tensile strength (BTS) values for 166 BC_1_F_2_ individuals between *Oryza* *sativa* IR36 and IL(*qSH3*-Npb, *sh4*-Npb), used for QTL-seq analysis. The average BTS values for the parent lines and their F_1_ are shown with black triangles. IR36: 45.6 ± 16.8 gf (n = 4), the IL(*qSH3*-Npb, *sh4*-Npb): 12.2 ± 3.4 gf (n = 4), and F_1_: 19.9 ± 5.6 gf (n = 4) (mean ± SD). The DNA samples of BC_1_F_2_ plants with BTS values between 11.8 and 16.2 gf were selected as low (L) bulk and those with BTS values between 44.7 and 66.0 gf were selected as high (H) bulk for further studies. The BTS values for each BC_1_F_2_ individual are shown in Supplementary Table 3

We previously observed segregation in the degree of seed shattering in an F_2_ population between IR36 and IL(*qSH3*-Npb, *sh4*-Npb). However, due to differing heading dates and morphological traits, proper evaluation of the degree of seed shattering was difficult (Tsujimura et al. 2017). To avoid segregation of other traits, we backcrossed an F_1_ plant with IR36 (Supplementary Fig. 1). A total of 116 BC_1_F_1_ plants were obtained with heading dates ranging from 1 to 18 August 2017 (Supplementary Table 2). Approximately, a month after heading, we evaluated the seed-shattering degree by measuring the BTS value for grain detachment of each BC_1_F_1_ plant, and the resulting values ranged from 18.8 gf to 37.9 gf (Fig. 2). Among these plants, we selected No. 112, as it had a low BTS value (19.7 gf). We expected the lower BTS value of the plant to be attributed to the W630-dominant allele(s) at the novel seed-shattering loci. The BTS values of the resulting BC_1_F_2_ plants were further evaluated for their seed-shattering degree. The BTS values of BC_1_F_2_ plants showed contentious segregation, with BTS values mostly falling between those of their parents: IL(*qSH3*-Npb, *sh4*-Npb), 12.2 ± 3.4 gf; and IR36, 45.6 ± 16.8 gf (Fig. 3; Supplementary Table 3). Because the *qSH3* and *sh4* alleles are fixed homozygously with domesticated alleles, the segregation of the degree of seed shattering in this population was likely caused by novel seed-shattering loci.

### Detection of novel loci involved in non-seed-shattering behaviour of IR36 by QTL-seq analysis

**Fig. 4:**
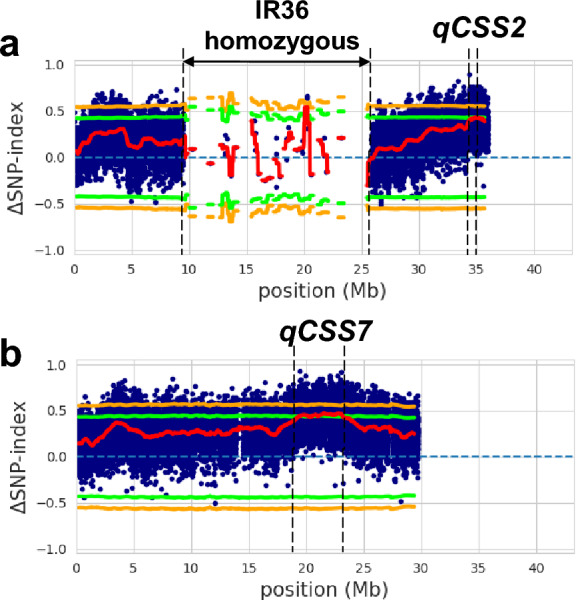
Detection of novel loci for seed shattering on chromosomes 2 (*qCSS2*) and 7 (*qCSS7*) by QTL-seq analysis. *qCSS2* and *qCSS7* were detected in approximately the 0.13-Mb and 3.73-Mb regions on chromosomes 2 (**a**) and 7 (**b**), respectively. The Δ(SNP-index) plots with statistical intervals under the null hypothesis of no QTL (orange, *P* < 0.01; green; *P* < 0.05) are shown. The red line indicates the average Δ (SNP-index) calculated by a sliding window analysis

To estimate the genetic region(s) underlying differences in the seed-shattering degree between IR36 and IL(*qSH3*-Npb, *sh4*-Npb), we subjected their BC_1_F_2_ plants for QTL-seq analysis (Takagi et al. 2013; Sugihara et al. 2022). A total of 100.3, 105.7, 56.1, and 51.0 million sequence reads (each 100 bp) were obtained from the DNA of H-bulk, L-bulk, IL, and IR36, respectively. Using the Illumina short read data, we first estimated the chromosomal segments of Nipponbare that were introgressed in the wild rice, *O. rufipogon* W630 genome. In IL(*qSH3*-Npb, *sh4*-Npb), we found the chromosomal segments on chrs. 1, 3, 4, 5, 6, 7, 11, and 12 were derived from Nipponbare (Supplementary Table 4). We also investigated the IR36-homozygously fixed chromosomal regions in the BC_1_F_2_ population associated with backcrossing (Supplementary Table 5), suggesting that these regions are unlikely to be responsible for the difference in the seed-shattering degree of the BC_1_F_2_ population. According to the introgressed chromosomal segments of Nipponbare in IL(*qSH3*-Npb, *sh4*-Npb), those on chromosomes 1 and 12 were not transmitted because of IR36-backcrossing, while partial or whole segments on chromosomes 3, 4, 5, 6, 7 and 11 were transmitted to BC_1_F_2_ population (Supplementary Tables 3 and 4). Notably, the Nipponbare segment on chromosome 4 was present in BC_1_F_2_ population, but the *sh4* genotype was fixed with domesticated allele of Nipponbare and IR36, that are identical (Fig. 1a). By examining the Δ(SNP-index) plot, we detected the two genomic regions with averaged Δ(SNP-index) values exceeding the 95% confidence interval by a sliding window analysis (statistical significance under the null hypothesis: *P* < 0.05): the regions on chr. 2 from 34.82 to 34.95 Mb with maximum Δ(SNP-index) = 0.43 (statistical significance under the null hypothesis: *P* < 0.05), and on chr. 7 from 19.43 to 23.15 Mb with maximum Δ(SNP-index) = 0.48 (Fig. 4). These two loci were named as *QTL for the Control of Seed Shatteringin rice on chromosomes 2 and 7* (*qCSS2* and *qCSS7*, respectively). Other regions exceeding the confidence interval were not detected in this analysis (Supplementary Fig. 2). Since the two regions were detected based on the segregation of IR36 and W630 chromosomal segments (Supplementary Tables 3 and 4), we estimated the *qCSS2* and *qCSS7* were likely to contribute to reduced seed-shattering behaviour in IR36.

To further confirm the results of QTL-seq analysis, genotypes at *qCSS2* and *qCSS7* regions were surveyed for each BC_1_F_2_ plant using DNA markers flanking the regions on chromosomes 2 or 7, respectively (Supplementary Table 1). Regarding *qCSS2*, the region estimated by QTL-seq analysis was between Indel2-3 and RM208. Genotyping analysis showed that 40 (24.1%) and 39 (23.5%) of BC_1_F_2_ plants carried the IR36 and W630 homozygous chromosomal segments, respectively (Supplementary Table 3). A comparison of the BTS values of the two homozygous groups based on the chromosomal constitutions showed that plants with the IR36 chromosomal segment tended to have higher BTS values than those with W630 (Supplementary Fig. 3). Similarly, we analysed the *qCSS7* region and found that 15 (9.0%) and 22 (13.3%) of BC_1_F_2_ plants carried IR36 and W630 homozygous chromosomal segments, respectively. We also found that plants with IR36 chromosomal segments at *qCSS7* region tended to exhibit higher BTS values than those with W630 (Supplementary Fig. 3). These surveys of the BTS values of each BC_1_F_2_ plant confirmed that both *qCSS2* and *qCSS7* regions were likely to be involved in the non-seed-shattering behaviour of IR36.

### Validation of the effect of *qCSS2* and *qCSS7* on seed-shattering degree

**Fig. 5:**
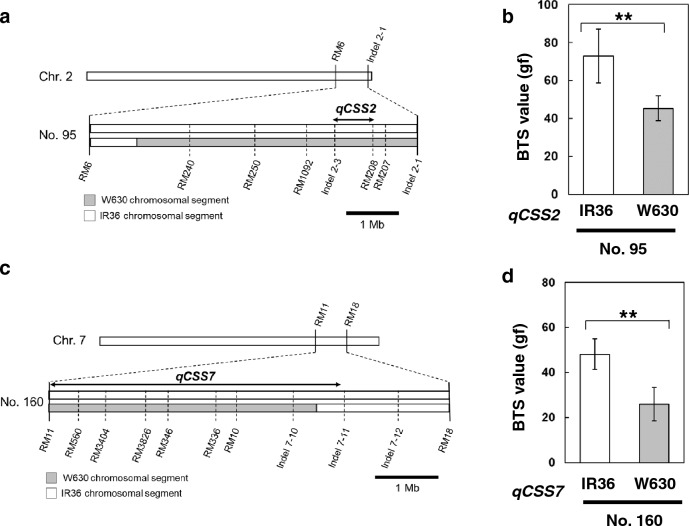
Validation of the effect of *qCSS2* and *qCSS7* on seed shattering by subsequent progeny test. **a** Progeny test of *qCSS2*. Graphical genotype of BC_1_F_3_ plants (No. 95). **b** Comparison of the average breaking tensile strength (BTS) values for the BC_1_F_3_ plants are shown. **c** Progeny test of *qCSS7*. Graphical genotype of BC_1_F_3_ plants (No. 160). **d** Comparison of the average BTS values for the BC_1_F_3_ plants are shown. Data are mean ± SD (n = 3–5). Estimated regions of *qCSS2* and *qCSS7* are shown based on the result of QTL-seq analysis, with the 95% confidence intervals. Genotypes at *qCSS2* and *qCSS7* regions were estimated by the corresponding DNA markers, and the average of the BTS values were calculated. ** indicates *P* < 0.01 by unpaired Student’s *t*-test

To further confirm the effect of the two loci on seed shattering, we carried out a progeny test for both the *qCSS2* and *qCSS7* loci. Two BC_1_F_2_ lines that carrying heterozygous chromosomal constitutions covering *qCSS2* or *qCSS7* candidate regions were selected (No. 95 for *qCSS2* and No. 160 for *qCSS7*). Their self-pollinated seeds of BC_1_F_3_ generation were germinated and then their genotypes were confirmed to have plants as one of the two loci fixed with the IR36 chromosomal segment based on the DNA markers in the region (Supplementary Table 1). For *qCSS2*, a progeny test using BC_1_F_3_ plants from No. 95 showed that plants with the IR36 chromosomal segment had a BTS of 73.0 ± 14.2 gf, while those with the W630 chromosomal segment had a BTS of 45.3 ± 6.5 gf, with a significant difference in their BTS values (Fig. 5a and b). Similarly, the effect of the *qCSS7* region on seed shattering was analysed using the progenies of two BC_1_F_2_ plants (No. 160). Difference in the BTS values was detected between plants carrying the IR36 and W630 chromosomal segments covering the *qCSS7* region (Fig. 5c and d). Taken together, the progeny test for *qCSS2* and *qCSS7* indicated that these two loci play important roles in controlling the seed-shattering degree in rice.

### Genetic interaction between *qCSS2* and *qCSS7* on reducing the degree of seed shattering in wild rice genetic background

**Fig. 6:**
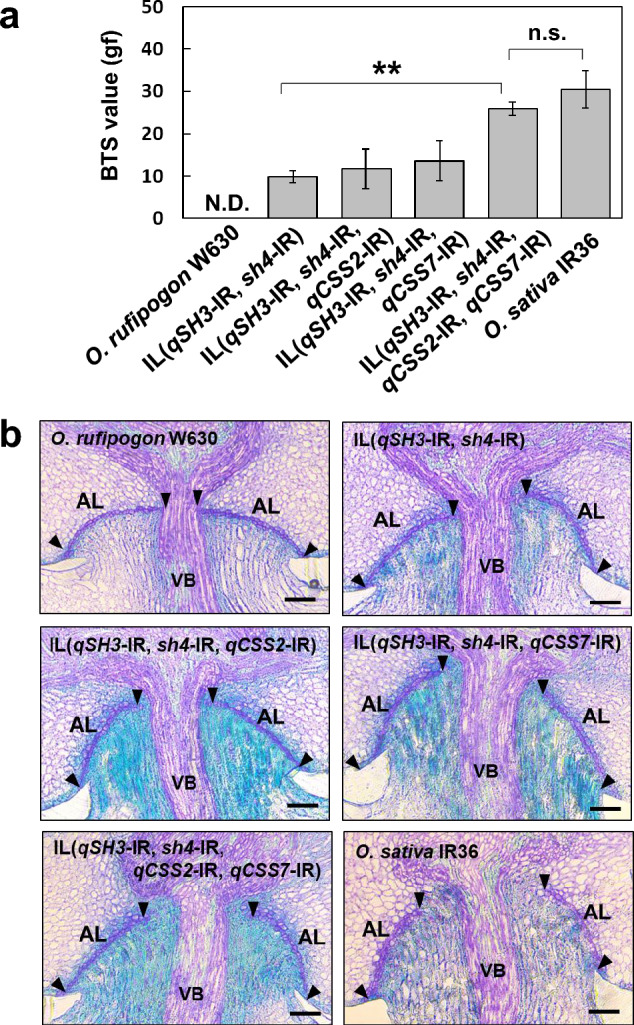
Evaluation of the genetic effect of the IR36 alleles at *qCSS2* and *qCSS7* on seed-shattering degree in the genetic background of wild rice, *O. rufipogon* W630. **a** Evaluation of seed-shattering degree by BTS values in the ILs of *qCSS2* and *qCSS7* with *qSH3* and *sh4*. ** and n.s. indicate significance (*P* < 0.01) and not significant (*P* ≥ 0.05) by unpaired Student’s *t*-test, respectively. N.D. Not determined owing to complete seed shattering. **b** Longitudinal sections of the spikelet base after seed detachment in W630, and the four ILs, and IR36. *VB* vascular bundle. *AL* abscission layer. Black triangles indicate both edges of the abscission layer. Scale bars = 50 μm. *O. rufipogon* W630 and *O. sativa* IR36 were used as the control

To further confirm the effect of the *qCSS2* and *qCSS7* loci on reducing the degree of seed shattering during rice domestication, we produced ILs that carry IR36 chromosomal segments covering *qCSS2* and *qCSS7* under the presence of *qSH3* and *sh4* mutations in the genetic background of wild rice, *O. rufipogon* W630. We previously evaluated backcrossed recombinant inbred lines (BRILs) between IR36 and *O. rufipogon* W630 (Ishikawa et al. 2017). Among these, we found that two independent lines, namely BRIL-A4 and BRIL-H6, sustained more grains than the other lines (Supplementary Fig. 4). We previously genotyped them at known seed-shattering loci of *qSH3* and *sh4*, finding that both carried IR36 domesticated alleles (Ishikawa et al. 2017). Interestingly, we noticed that the A4 and H6 lines contained IR36 chromosomal segments covering the *qCSS2* and *qCSS7* regions, respectively (Supplementary Fig. 4), suggesting that these two loci may contribute to further reductions in seed shattering of the two lines. To understand the genetic interaction between these two loci under the presence of *qSH3* and *sh4* mutations, we crossed these two lines, and the resulting F_1_ plants were self-pollinated to obtain F_2_ seeds. We employed two SSR markers for each of *qCSS2* and *qCSS7* to investigate the chromosomal constitutions covering the two loci. Based on the genotypes of the two loci, we produced four ILs: namely IL(*qSH3*-IR, *sh4*-IR), IL(*qSH3*-IR, *sh4*-IR, *qCSS2*-IR), IL(*qSH3*-IR, *sh4*-IR, *qCSS7*-IR), and IL(*qSH3*-IR, *sh4*-IR, *qCSS2*-IR, *qCSS7*-IR) in wild rice genetic background of *O. rufipogon* W630 (Supplementary Fig. 5). Seeds retained in the panicles of the four lines tended to be greater as the number of IR-chromosomal segments at *qCSS2* and *qCSS7* increased (Supplementary Fig. 6). We measured their BTS values together with the controls of *O. rufipogon* W630 and *O. sativa* IR36 (Fig. 6a). A proportional increase in the BTS values was observed depending on the number of seed-shattering loci with domesticated alleles (Fig. 6a). The BTS values for IL(*qSH3*-IR, *sh4*-IR) was 9.8 ± 1.4 gf. A slight increase in the BTS values of 11.7 ± 4.7 gf and 13.6 ± 4.7 gf were observed for IL(*qSH3*-IR, *sh4*-IR, *qCSS2*-IR) and IL(*qSH3*-IR, *sh4*-IR, *qCSS7*-IR), respectively, while significantly larger BTS values of 25.9 ± 1.6 gf were obtained for IL(*qSH3*-IR, *sh4*-IR, *qCSS2*-IR, *qCSS7*-IR). We then carried out a two-way analysis of variance (ANOVA) to investigate the relationship between *qCSS2* and *qCSS7* and found a significant digenic interaction (*P* < 0.05). Interestingly, no significant difference was detected between IL(*qSH3*-IR, *sh4*-IR, *qCSS2*-IR, *qCSS7*-IR) and IR36 (30.5 ± 4.4 gf) (Fig. 6a). We also analysed abscission layer formation for these ILs together with *O. rufipogon* W630 and *O. sativa* IR36. We found a proportional increase in the inhibition of abscission layer formation that was correlated with the BTS values (Fig. 6b). These results imply that the non-seed-shattering behaviour of IR36 can be controlled by the variations at least four seed-shattering loci: *qSH3*, *sh4*, *qCSS2*, and *qCSS7*.

## Discussion

### Genetic dissection of quantitatively controlled non-seed-shattering behaviour of an *indica* rice cultivar, IR36

As Nipponbare and IR36 share the same alleles at *qSH3* and *sh4*, the reduced seed-shattering characteristics of IR36 cannot be fully explained by these two alleles alone. Based on this observation, we conducted a genetic analysis to detect additional factor(s) contributing to the reduced seed-shattering behaviour of IR36. By backcrossing the F_1_ plant between IR36 and IL(*qSH3*-Npb, *sh4*-Npb), we first evaluated their BC_1_F_1_ population to screen the plants with the lowest BTS values. Based on the QTL-seq analysis using BTS values in the BC_1_F_2_ population, we identified two novel loci, *qCSS2* and *qCSS7*. The effect of these loci on seed shattering was evaluated in each plant of the BC_1_F_2_ population and subsequent progeny tests (Supplementary Fig. 3, Fig. 5), confirming their roles in controlling seed shattering.

To investigate the genetic basis of the non-seed shattering behaviour of IR36, we produced ILs between IR36 and W630. Among the BRILs produced previously, we selected those carrying domesticated alleles at *qSH3* and *sh4*, after screening their genotypes at these two loci (Ishikawa et al. 2017). Interestingly, the two lines exhibited relatively higher BTS values than those of the other lines. We noticed that these lines carried IR36 chromosomal segments in either the regions covering *qCSS2* or *qCSS7* (Supplementary Fig. 4). The high BTS values of these lines may be associated with *sh4*, *qSH3*, and *qCSS2*, or *qCSS7*. The BTS values of IL(*qSH3*-IR, *sh4*-IR, *qCSS2*-IR, *qCSS7*-IR) were significantly higher than those of IL(*qSH3*-IR, *sh4*-IR, *qCSS2*-IR) or IL(*qSH3*-IR, *sh4*-IR, *qCSS7*-IR), thereby confirming that the two loci play roles in reducing seed shattering (Fig. 6).

In the present study, we detected *qCSS2* on chromosome 2 (Figs. 4 and 5). This region is closely linked to *Sh13*, which locates between RM208 and RM207 and was originally identified as a major locus responsible for the difference in the degree of seed shattering between cv. Oonari and cv. Takanari, moderate and easy-shattering rice cultivars, respectively (Li et al. 2019). The tendency toward seed shattering of Takanari was improved by gamma-ray irradiation-mediated mutagenesis, and the plants with reduced seed shattering were screened. Genetic analysis identified a responsible locus containing *osa-mir172d* miRNA, which was previously shown to control the grain threshability of wheat (Debernardi et al. 2017). Furthermore, upregulation of the *mir172* miRNA is likely associated with gene duplication. The involvement of *osa-mir172d* miRNA is vital for understanding the regulation of seed shattering in rice, however, this mutation was obtained by artificially induced mutagenesis of gamma-ray irradiation (Li et al. 2019). A survey of the sequence of *osa-mir172d* in *O. rufipogon* W630, *O. sativa* Nipponbare, and IR36 showed that they share the same sequence for the primary transcript (Supplementary Fig. 7). Therefore, we posited here that *osa-mir172d* miRNA is unlikely to be involved in the difference of seed shattering degree in our segregating population, although further mapping experimentation is required to clarify the precise involvement of the *osa-mir172d* miRNA in *qCSS2*. Regarding *qCSS7*, a QTL named *sh7.1* was previously detected near RM214 (approximately 12.78 Mb) on chromosome 7 using an advanced backcross population between *O. rufipogon* and *O. sativa* cv. Jefferson (Thomson et al. 2003). However, the position of *qCSS7* (19.43 to 23.15 Mb by QTL-seq analysis) is far from *sh7.1.* As there are no previously known seed-shattering loci near *qCSS7*, novel factor(s) may underlie this region. Accordingly, further genetic dissections at the two loci are required to narrow down the candidate regions for identifying the causal mutations at *qCSS2* and *qCSS7*. Taken together, *qCSS2* and *qCSS7* may encode novel genes or mutations distinct from those induced artificially.

### Common and distinct loci involved in non-seed-shattering behaviour in *japonica* and *indica* rice cultivars

In this study, we detected two novel loci, *qCSS2* and *qCSS7*, involved in reduced seed-shattering behaviour of an *indica* rice cultivar IR36 (Fig. 4). In contrast, we previously detected *qCSS3*, a novel locus involved in the reduced seed-shattering behaviour of the *japonica* rice cultivar *O. sativa* Nipponbare (Tsujimura et al. 2019). The *qCSS3* was detected by QTL-seq analysis of F_2_ plants between Nipponbare and the IL(*qSH1*-Npb, *sh4*-Npb, *qSH3*-Npb), which carries Nipponbare chromosomal segments covering *qSH1*, *sh4*, and *qSH3* regions in the genetic background of wild rice, *O. rufipogon* W630. In our QTL-seq analysis of reduced-seed-shattering behaviour in IR36, the *qCSS3* region, located ~ 8.30 to 13.65 Mb, was not detected (Supplementary Fig. 2). This suggests that *qCSS3* is unlikely to be involved in the reduced seed-shattering behaviour of IR36. In addition, *qSH1* has also been detected as a locus involved in the reduction of seed shattering in some *japonica* rice cultivars (Konishi et al. 2006). As previously reported, the *qSH1* malfunction allele is not observed in *indica* rice cultivars (Zhang et al. 2009), it is specific to *japonica* rice cultivars. Regarding our previous QTL-seq analysis of the non-seed-shattering behaviour of Nipponbare, no peaks associated with seed shattering were observed in either the *qCSS2* or *qCSS7* regions (Tsujimura et al. 2019), suggesting that the two loci detected in this study are unlikely to be involved in the control of seed shattering in *japonica* rice cultivars.

Asian rice is largely classified into two groups: *indica* and *japonica*. In both subspecies, two seed-shattering loci of *qSH3* and *sh4* are commonly involved in reducing seed shattering, while those at *qSH1* and *qCSS3* or *qCSS2* and *qCSS7* are specific to their cultivar group. Interestingly, *circum*-aus rice cultivars carry a functional (non-domesticated) allele at *qSH3* (Ishikawa et al. 2022). This finding is of particular interest since we do not know whether the seed-shattering loci detected using Nipponbare and IR36 are also involved in reduced seed-shattering behaviour for *circum-*aus rice cultivars. Once the causal mutations at the *qCSS2*, *qCSS3*, and *qCSS7* loci are identified, the survey of mutations at these loci for *indica*, *japonica*, and *circum-*aus cultivars will provide an important scope for understanding the genetic basis of reduced seed shattering in rice. Our findings imply that a distinct history of selections may underlie the process of rice domestication. Seed-shattering loci with specific roles can be important for controlling seed shattering in cross-breeding programmes among rice cultivars with diverse origins, and are also informative in clarifying the processes of rice domestication.

### Quadratic increase in BTS followed by integration of domesticated alleles at seed-shattering loci

We previously reported that a single mutation at one of the known seed-shattering loci was not sufficient to inhibit abscission layer formation in wild rice genetic background (Ishii et al. 2013, Htun et al. 2014; Inoue et al. 2015). Accordingly, further investigation of the individual effects of *qCSS2* or *qCSS7* domesticated alleles alone on the loss of seed shattering is necessary to confirm their effect in wild rice. Notably, these regions have not been previously studied in the genetic analyses of seed shattering in rice, and it is likely that their effects are smaller than those of major loci, thus, an effect from either single mutation may not be observed in wild rice. In IL(*qSH3*-IR, *sh4*-IR), a slight inhibition of the abscission layer was observed around the central vascular bundles (Fig. 6b). Additionally, the IL(*qSH3*-IR, *sh4*-IR, *qCSS2*-IR) or IL(*qSH3*-IR, *sh4*-IR, *qCSS7*-IR), which have an IR36 chromosomal segment at both of *qSH3* and *sh4* and either of *qCSS2* or *qCSS7* in the wild genetic background, resulted in increased inhibition of the abscission layer from the central vascular bundles (Fig. 6b). This increase in abscission layer inhibition was associated with a slight increase in the BTS values (Fig. 6a). A large increase in the BTS value was observed for plants with quadruple introgressions; IL(*qSH3*-IR, *sh4*-IR, *qCSS2*-IR, *qCSS7*-IR). Compared to IL(*qSH3*-IR, *sh4*-IR, *qCSS2*-IR) or IL(*qSH3*-IR, *sh4*-IR, *qCSS7*-IR), a further increase in the length of inhibited region in abscission layer in IL(*qSH3*-IR, *sh4*-IR, *qCSS2*-IR, *qCSS7*-IR) in vertical sections was similar to that caused by the additional effects of *qCSS2* or *qCSS7* with *qSH3* and *sh4* (Fig. 6b). The notably high BTS value of IL(*qSH3*-IR, *sh4*-IR, *qCSS2*-IR, *qCSS7*-IR) can be explained by the inhibited area of the abscission layer, as the area of abscission layer inhibition can be calculated using the radius squared (Ishikawa et al. 2022). These results suggest that the quadratic increase in the BTS values with the accumulation of the domesticated alleles at seed-shattering loci may be the genetic basis of the reduced seed-shattering behaviour of cultivated rice. This increase in the degree of seed shattering may be associated with the development of harvesting tools during domestication. In the early stages of domestication, primitive tools were ideal for harvesting, whereas more advanced tools were employed in the later eras. Evaluating the effect of common and subspecies-specific seed-shattering loci that are associated with reduced seed shattering will be particularly informative in understanding the process of rice domestication in terms of phenotypic changes to rice and thus the development of human civilisation.

## Supplementary Information

Below is the link to the electronic supplementary material.

Supplementary file1 (PDF 1359 KB)

Supplementary file2 (XLSX 32 KB)

## Data Availability

Raw fastq reads for High-bulk, Low-bulk, IL(*qSH3*-Npb, *sh4*-Npb), and IR36 were deposited in the Sequence Read Archive (SRA) under DRA accession number DRA016170.
