# Correction: Detection of novel loci involved in non-seed-shattering behaviour of an *indica* rice cultivar, *Oryza sativa* IR36

**DOI:** 10.1007/s00438-023-02047-9

**Published:** 2023-06-23

**Authors:** Shohei Sugiyama, Motoki Sakuta, Yuki Tsujimura, Yudai Yamaguchi, Than Myint Htun, Chizuru Inoue, Koji Numaguchi, Takashige Ishii, Ryo Ishikawa

**Affiliations:** 1 Laboratory of Plant Breeding, Graduate School of Agricultural Science, Kobe University, 1-1 Rokkodai-cho, Nada-ku, Kobe, 657-8501 Japan; 2 Present Address: Department of Plant Breeding, Physiology and Ecology, Yezin Agricultural University, Yezin, Nay Pyi Taw, 15013 Myanmar

**Correction: Molecular Genetics and Genomics (2023) 298:943–953** 10.1007/s00438-023-02027-z

In the published article, in this article reference Ishii et al. 2013 was processed with error and the correct reference should have been

‘Ishii T, Numaguchi K, Miura K, Yoshida K, Thanh PT, Htun TM, Yamasaki M, Komeda N, Matsumoto T, Terauchi R, Ishikawa R, Ashikari M (2013) OsLG1 regulates a closed panicle trait in domesticated rice. Nat Genet 45:462–465. https://doi.org/10.1038/ng.2567’.

The original article has been updated.

