# Supplementary material for: Detection of novel loci involved in non-seed-shattering behaviour of an indica rice cultivar, Oryza sativa IR36
Source: Mol Genet Genomics. 2023 May 17;298(4):943–53. doi: 10.1007/s00438-023-02027-z (PMC10227132; doi:10.1007/s00438-023-02027-z)
Supplement: Supplementary file 1 — Supplementary file1 (PDF 1359 KB) [file 438_2023_2027_MOESM1_ESM.pdf]

Sugiyama and Sakuta et al. Supplementary Figure 1

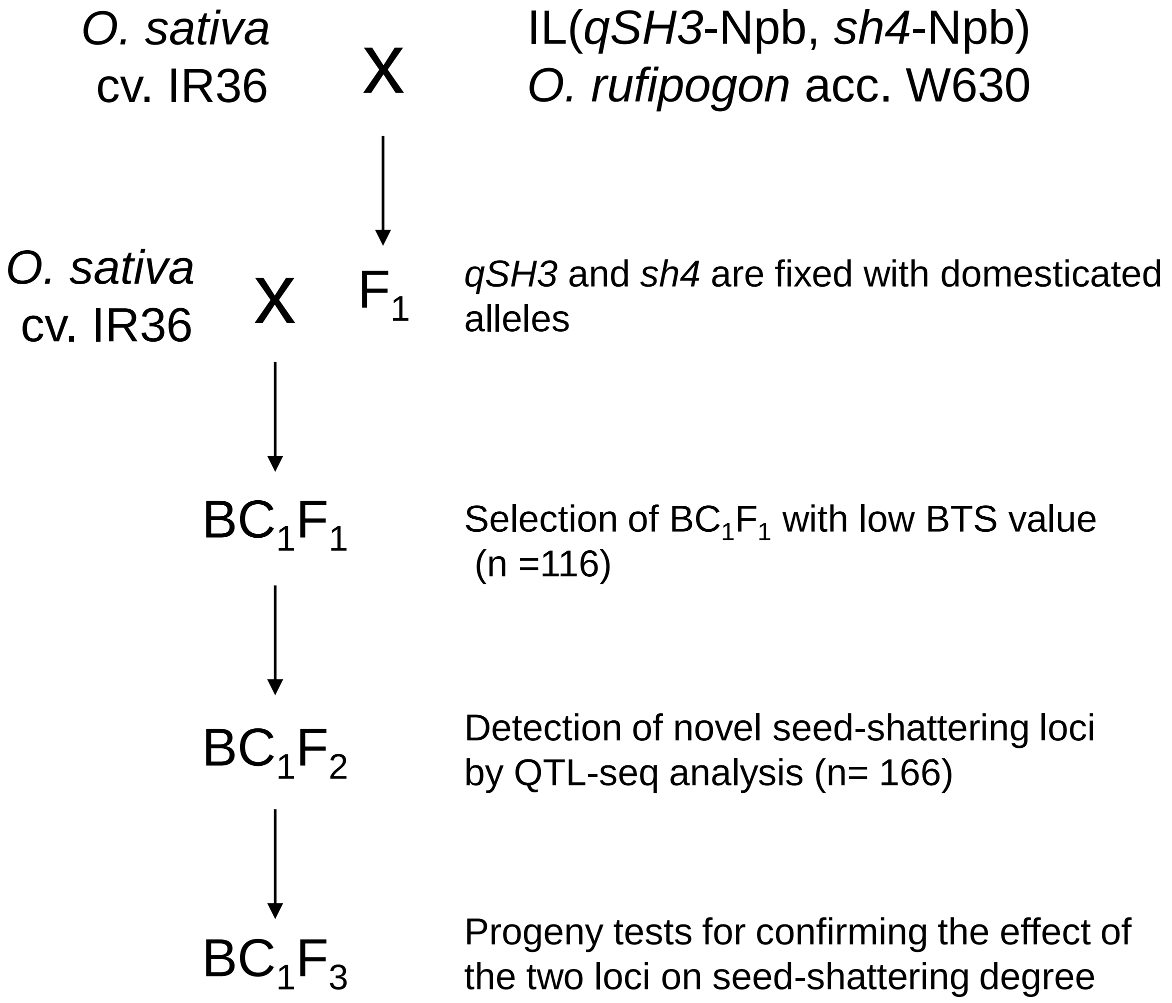

Supplementary Figure 1. Pedigree of backcross populations between *O. sativa* IR36 and *O. rufipogon* W630. An introgression line, IL(*qSH3*-Npb, *sh4*-Npb) carrying Nipponbare chromosomal segments covering the two loci, was crossed with IR36, and a resulting F_1 plant was further backcrossed with IR36. Among 116 BC_1F_1 plants, one line, No.112, was selected to obtain BC_1F_2 segregating population.

Sugiyama and Sakuta et al. Supplementary Figure 2

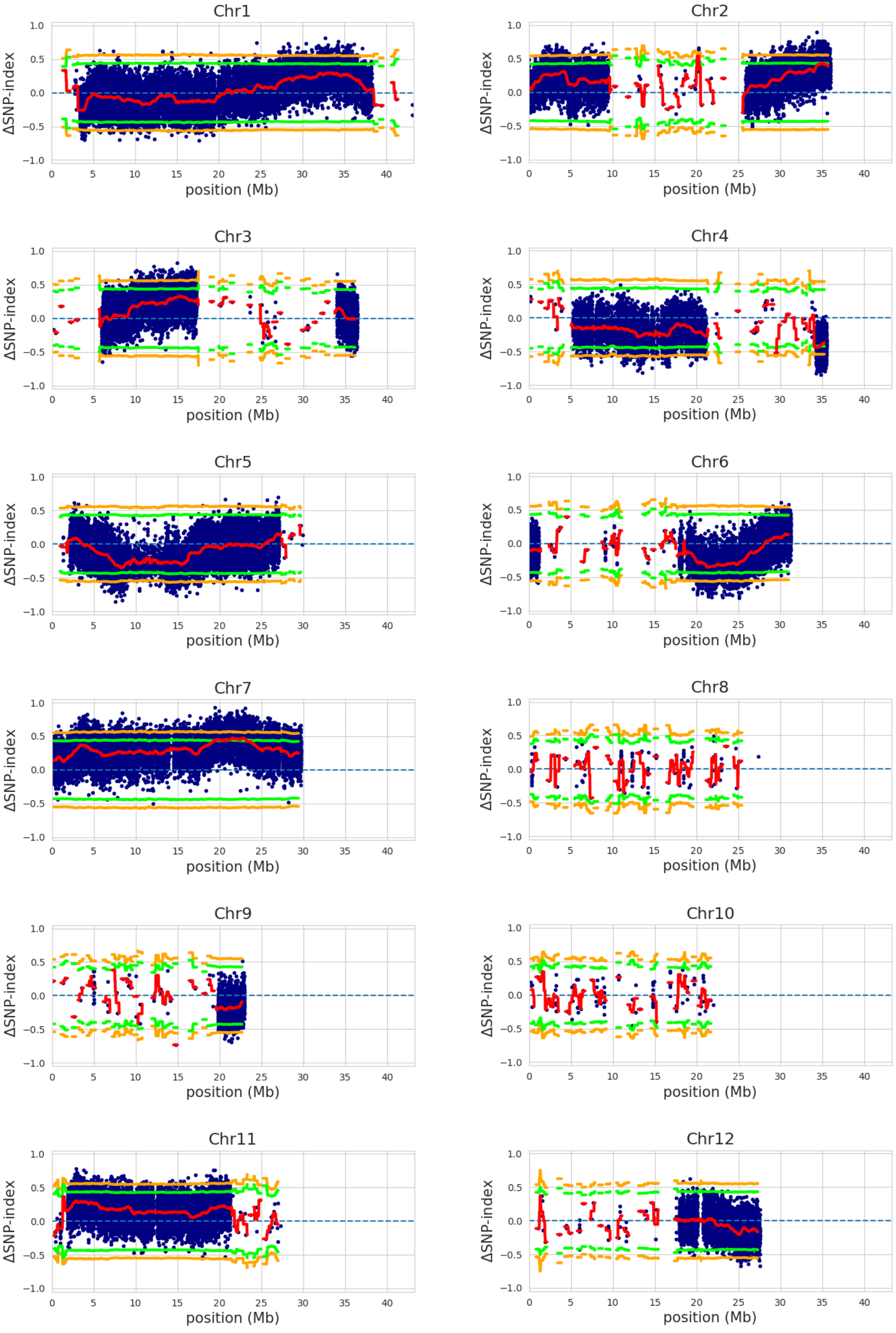

Supplementary Figure 2. Results of the QTL-seq analysis for all chromosomes. The Δ(SNP-index) plots with statistical intervals under the null hypothesis of no QTL (orange, $P < 0.01$; green; $P < 0.05$). No.112 was selected to obtain BC_1F_2 segregating population.

Sugiyama and Sakuta et al. Supplementary Figure 3

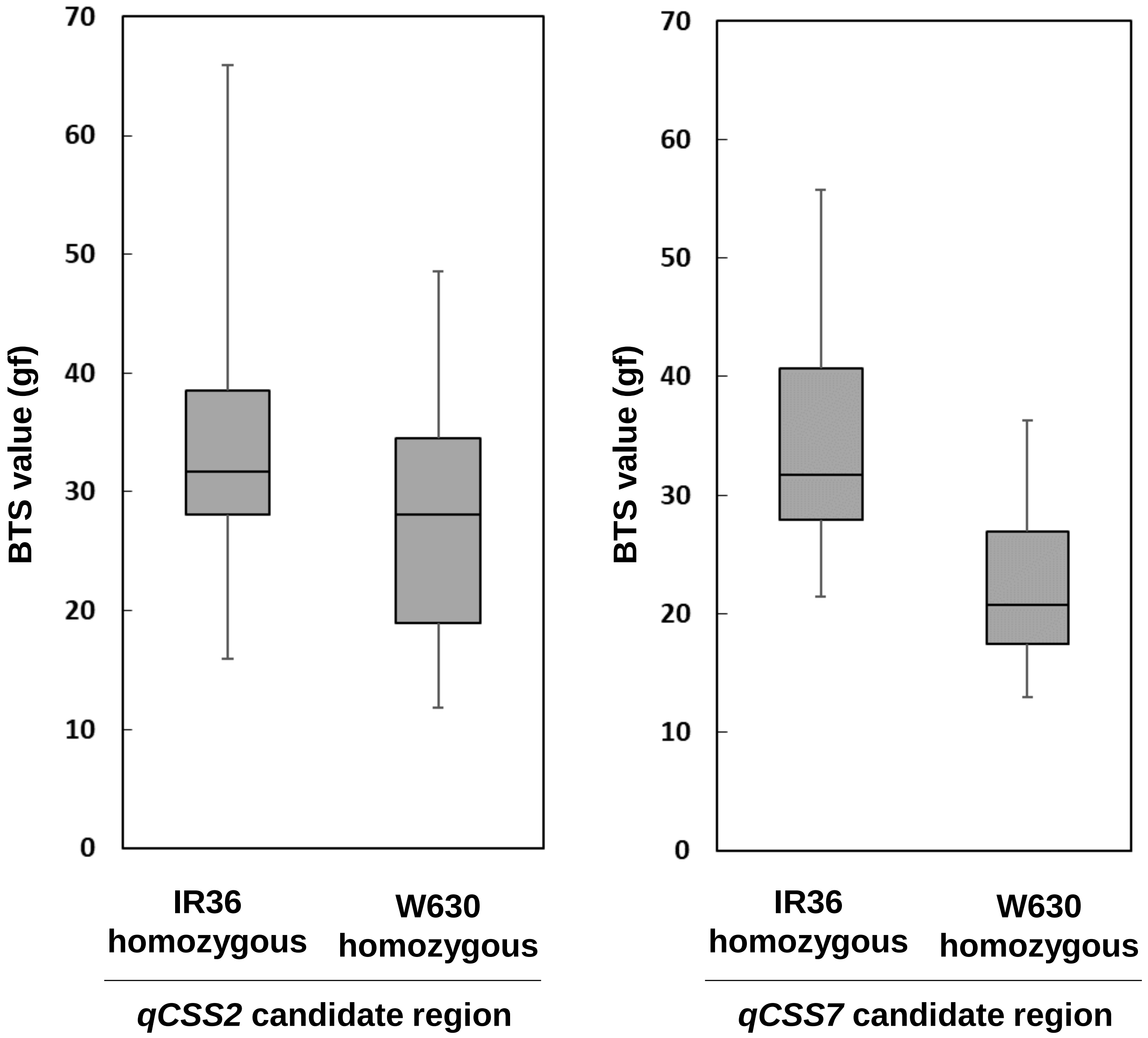

Supplementary Figure 3. Box plots of the BTS values of two BC_1F_2 groups based on the genotypes at the candidate regions of *qCSS2* and *qCSS7*. All BC_1F_2 individuals were surveyed with two and three DNA markers covering *qCSS2* and *qCSS7* region, respectively (Supplementary Tables 1 and 2). Plants carrying the IR36 and W630 homozygous chromosomal segments for the entire candidate regions of *qCSS2* and *qCSS7* were selected.

Sugiyama and Sakuta et al. Supplementary Figure 4

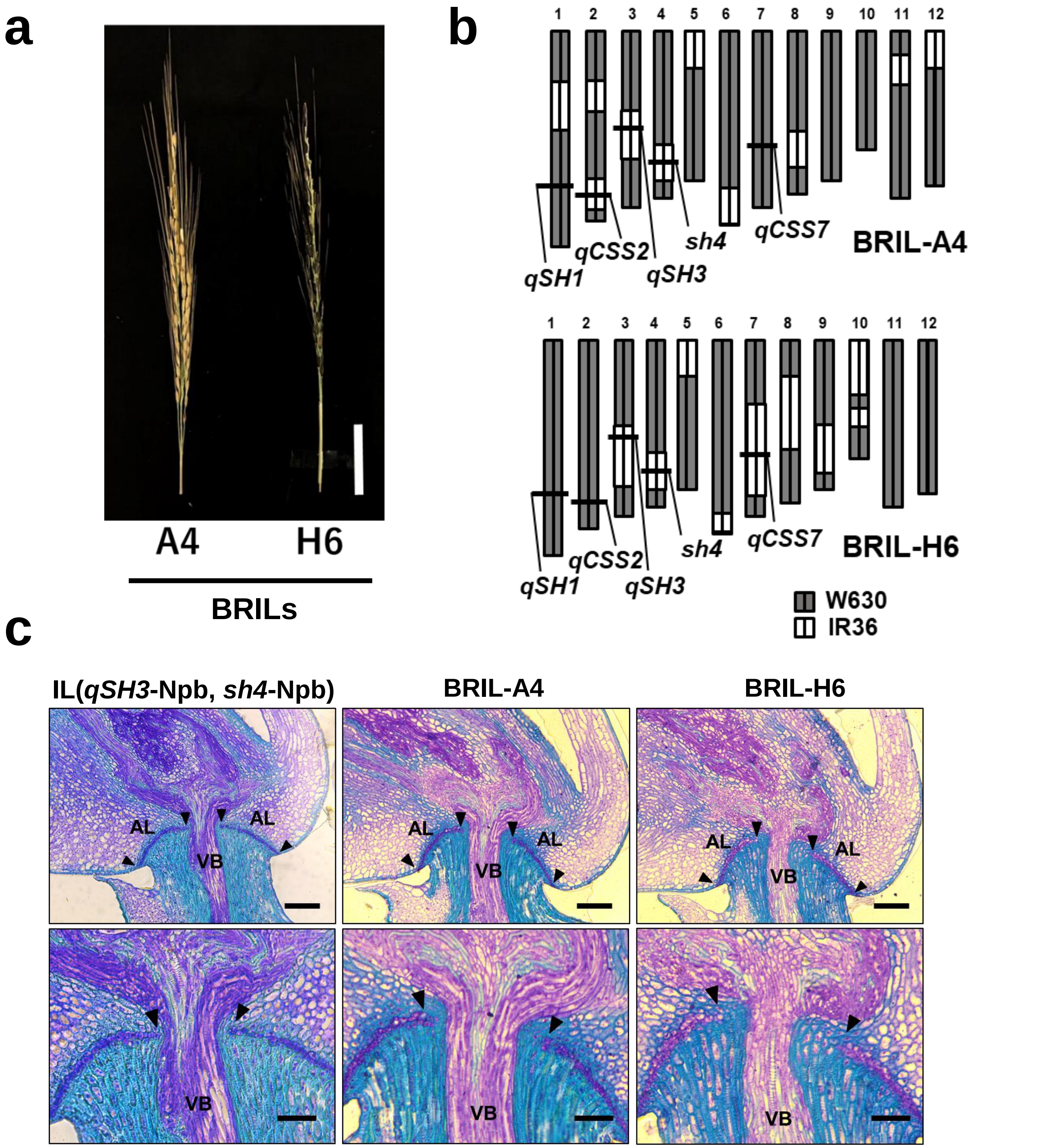

Supplementary Figure 4. Characterisation of two backcross recombinant inbred lines (BRILs) between *O. sativa* IR36 and *O. rufipogon* W630.
(a) Panicle photo of two BRILs namely A4 and H6 at approximately a month after flowering. Bar 5 cm. (b) Graphical genotype of two BRILs of A4 (upper) and H6 (lower). (c) Abscission layer formation of two BRILs of A4 (centre) and H6 (right). IL(*qSH3*-Npb, *sh4*-Npb) are shown as a control (left). VB, vascular bundle. AL, abscission layer. Black triangles indicate both edges of the abscission layer. Scale bars = 100 μm (upper panel), 50 μm (lower panel).

Sugiyama and Sakuta et al. Supplementary Figure 5

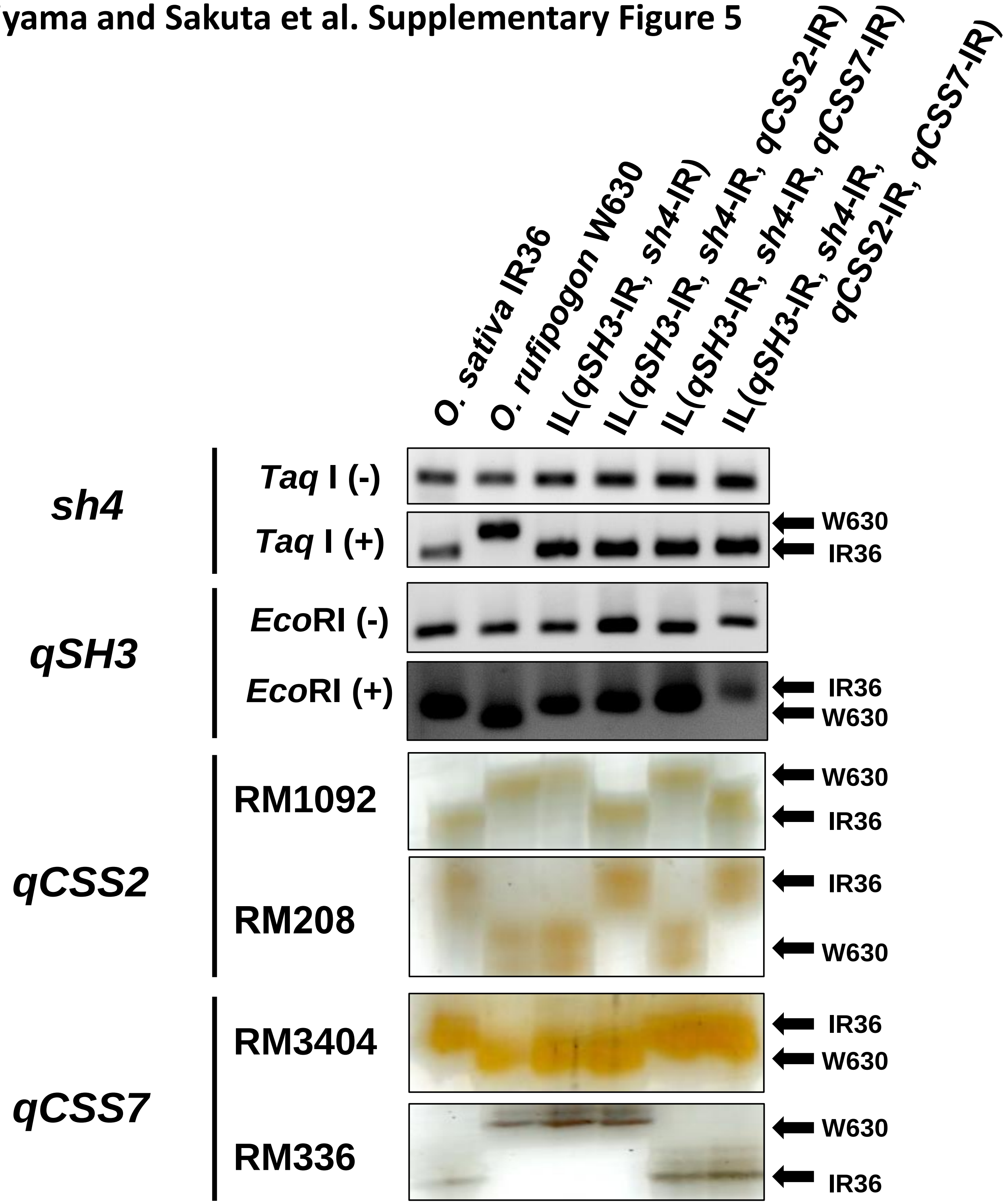

Supplementary Figure 5. Genotyping of the introgression lines (ILs) for *qSH3*, *sh4*, *qCSS2,* and *qCSS7* in the genetic background of wild rice, *O. rufipogon* W630. dCAPS markers were employed for genotyping of causal SNPs at *sh4* and *qSH3*, while SSR markers RM1092 / RM208 and RM3404 / RM336 were employed for genotyping at *qCSS2* and *qCSS7*, respectively. (-) and (+) indicate without and with restriction endonuclease. Arrows on right side of the gel photos indicate the alleles of W630 or IR36.

Sugiyama and Sakuta et al. Supplementary Figure 6

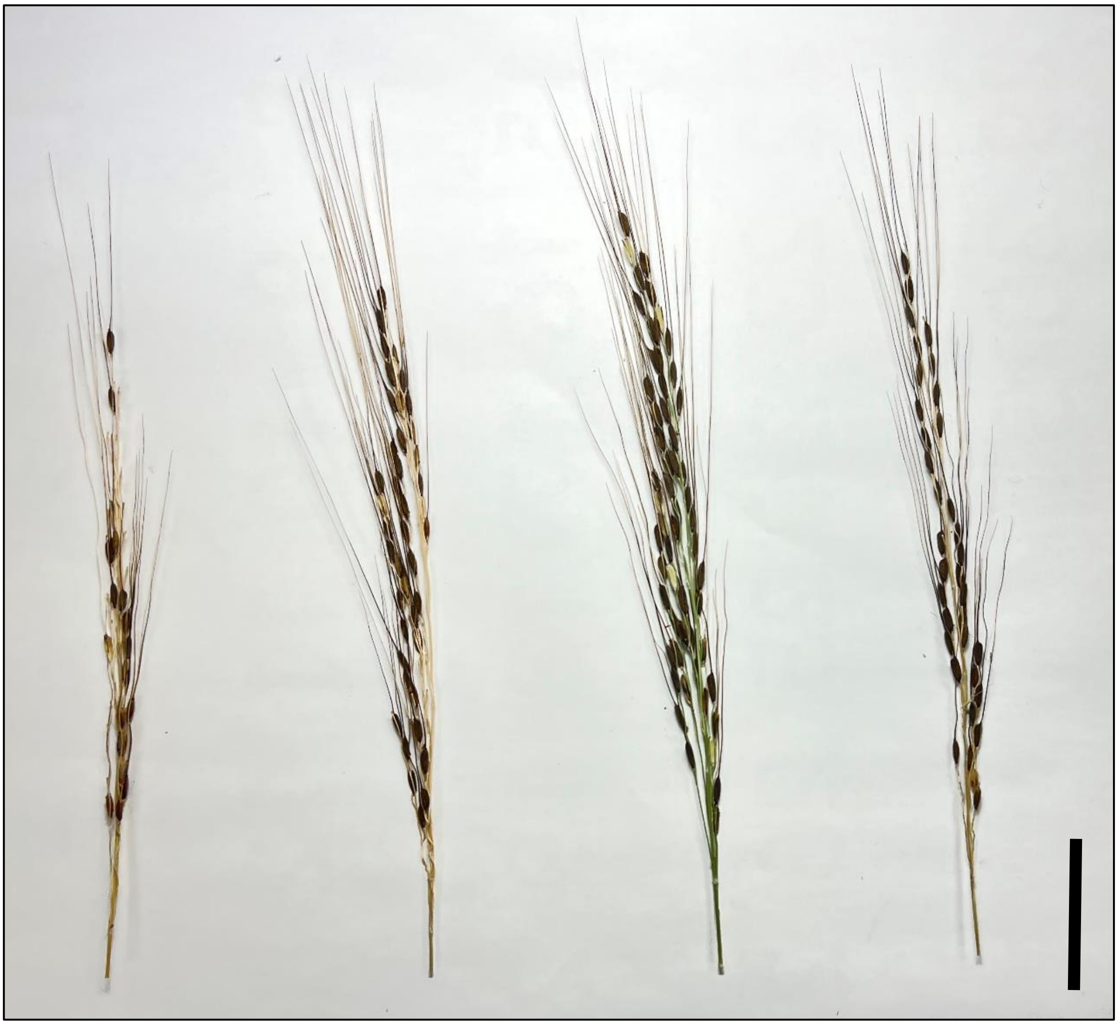

IL(*qSH3-IR*, *sh4-IR*)

IL(*qSH3-IR*, *sh4-IR*, *qCSS2-IR*)

IL(*qSH3-IR*, *sh4-IR*, *qCSS7-IR*)

IL(*qSH3-IR*, *sh4-IR*, *qCSS2-IR*, *qCSS7-IR*)

Supplementary Figure 6. Panicle photo of four introgression lines (ILs) generated from the cross between two backcross recombinant inbred lines, A4 and H6 at approximately a month after flowering. Scale bar = 5 cm.

Sugiyama and Sakuta et al. Supplementary Figure 7

```
W630       AAACAGTCGGTGCTTGCAGGTGCAGCACCATCAAGATTCACATCGAGTTCATCCCTAAAC
IR36       AAACAGTCGGTGCTTGCAGGTGCAGCACCATCAAGATTCACATCGAGTTCATCCCTAAAC
Npb        AAACAGTCGGTGCTTGCAGGTGCAGCACCATCAAGATTCACATCGAGTTCATCCCTAAAC
           ************************************************************

W630       GAGATCGAGGTTGGCTGACTATATGTGATGAGAATCTTGATGATGCTGCATCAGCAAACG
IR36       GAGATCGAGGTTGGCTGACTATATGTGATGAGAATCTTGATGATGCTGCATCAGCAAACG
Npb        GAGATCGAGGTTGGCTGACTATATGTGATGAGAATCTTGATGATGCTGCATCAGCAAACG
           ************************************************************

W630       CTCGACTACT
IR36       CTCGACTACT
Npb        CTCGACTACT
           **********
```

Supplementary Figure 7. Multiple alignment of the primary transcript of *osa-mir172d* in *O. rufipogon* W630, *O. sativa* IR36, and *O. sativa* Nipponbare (Npb).
